# Supplementary material for: Modeling glioblastoma heterogeneity as a dynamic network of cell states
Source: Mol Syst Biol. 2021 Sep 16;17(9):e10105. doi: 10.15252/msb.202010105 (PMC8444284; doi:10.15252/msb.202010105)
Supplement: Supplementary file 5 — Source Data for Figure 3 [file MSB-17-e10105-s001.zip › Figure3A_sourcedata/GSEA_3065/nosigdb_state2.GseaPreranked.1622718419215/FELDMAN_2020_G1.html]

Details for gene set FELDMAN\_2020\_G1[GSEA]

|  || Dataset | state2 |
| Phenotype | NoPhenotypeAvailable |
| Upregulated in class | na\_pos |
| GeneSet | FELDMAN\_2020\_G1 |
| Enrichment Score (ES) | 0.8674445 |
| Normalized Enrichment Score (NES) | 3.0289822 |
| Nominal p-value | 0.0 |
| FDR q-value | 0.0 |
| FWER p-Value | 0.0 |
Table: GSEA Results Summary

  

Fig 1: Enrichment plot: FELDMAN\_2020\_G1      
 Profile of the Running ES Score & Positions of GeneSet Members on the Rank Ordered List

  

| PROBE | GENE SYMBOL | GENE\_TITLE | RANK IN GENE LIST | RANK METRIC SCORE | RUNNING ES | CORE ENRICHMENT || 1 | HIST1H4C |  |  | 0 | 1.309 | 0.0220 | Yes |
| 2 | HIST1H1B |  |  | 1 | 0.902 | 0.0372 | Yes |
| 3 | HMGB2 |  |  | 4 | 0.823 | 0.0508 | Yes |
| 4 | TYMS |  |  | 5 | 0.792 | 0.0641 | Yes |
| 5 | CENPU |  |  | 6 | 0.763 | 0.0769 | Yes |
| 6 | H2AFZ |  |  | 8 | 0.735 | 0.0891 | Yes |
| 7 | UBE2C |  |  | 9 | 0.704 | 0.1010 | Yes |
| 8 | H2AFX |  |  | 10 | 0.675 | 0.1123 | Yes |
| 9 | DHFR |  |  | 11 | 0.644 | 0.1231 | Yes |
| 10 | UBE2T |  |  | 12 | 0.643 | 0.1339 | Yes |
| 11 | MCM7 |  |  | 13 | 0.626 | 0.1445 | Yes |
| 12 | TOP2A |  |  | 15 | 0.596 | 0.1544 | Yes |
| 13 | PCNA |  |  | 16 | 0.595 | 0.1644 | Yes |
| 14 | MKI67 |  |  | 17 | 0.590 | 0.1743 | Yes |
| 15 | CDK1 |  |  | 18 | 0.577 | 0.1840 | Yes |
| 16 | UBE2S |  |  | 19 | 0.574 | 0.1936 | Yes |
| 17 | MAD2L1 |  |  | 20 | 0.563 | 0.2031 | Yes |
| 18 | ATAD2 |  |  | 21 | 0.563 | 0.2125 | Yes |
| 19 | RRM2 |  |  | 22 | 0.556 | 0.2219 | Yes |
| 20 | CKS1B |  |  | 23 | 0.554 | 0.2312 | Yes |
| 21 | DUT |  |  | 24 | 0.552 | 0.2405 | Yes |
| 22 | HMGB1 |  |  | 25 | 0.549 | 0.2497 | Yes |
| 23 | NUDT1 |  |  | 26 | 0.541 | 0.2588 | Yes |
| 24 | CLSPN |  |  | 27 | 0.536 | 0.2678 | Yes |
| 25 | CKS2 |  |  | 28 | 0.532 | 0.2767 | Yes |
| 26 | TPX2 |  |  | 29 | 0.531 | 0.2857 | Yes |
| 27 | ESCO2 |  |  | 30 | 0.530 | 0.2946 | Yes |
| 28 | DEK |  |  | 31 | 0.526 | 0.3034 | Yes |
| 29 | STMN1 |  |  | 33 | 0.520 | 0.3120 | Yes |
| 30 | CENPF |  |  | 34 | 0.518 | 0.3207 | Yes |
| 31 | HMGN2 |  |  | 35 | 0.512 | 0.3293 | Yes |
| 32 | RPA3 |  |  | 36 | 0.511 | 0.3379 | Yes |
| 33 | GINS2 |  |  | 37 | 0.506 | 0.3464 | Yes |
| 34 | HIST1H1C |  |  | 38 | 0.498 | 0.3548 | Yes |
| 35 | SMC2 |  |  | 39 | 0.493 | 0.3631 | Yes |
| 36 | ORC6 |  |  | 40 | 0.493 | 0.3714 | Yes |
| 37 | DBF4 |  |  | 41 | 0.492 | 0.3796 | Yes |
| 38 | SMC4 |  |  | 42 | 0.489 | 0.3879 | Yes |
| 39 | TMPO |  |  | 43 | 0.489 | 0.3961 | Yes |
| 40 | CENPK |  |  | 44 | 0.488 | 0.4043 | Yes |
| 41 | ZWINT |  |  | 45 | 0.488 | 0.4125 | Yes |
| 42 | PBK |  |  | 46 | 0.484 | 0.4206 | Yes |
| 43 | NUSAP1 |  |  | 47 | 0.483 | 0.4287 | Yes |
| 44 | ANP32B |  |  | 48 | 0.474 | 0.4367 | Yes |
| 45 | PTMA |  |  | 49 | 0.474 | 0.4446 | Yes |
| 46 | KPNA2 |  |  | 50 | 0.472 | 0.4526 | Yes |
| 47 | TUBA1B |  |  | 52 | 0.462 | 0.4602 | Yes |
| 48 | CENPM |  |  | 54 | 0.460 | 0.4678 | Yes |
| 49 | TK1 |  |  | 56 | 0.456 | 0.4754 | Yes |
| 50 | NCAPG |  |  | 60 | 0.445 | 0.4826 | Yes |
| 51 | BIRC5 |  |  | 61 | 0.443 | 0.4900 | Yes |
| 52 | SIVA1 |  |  | 62 | 0.439 | 0.4974 | Yes |
| 53 | PTTG1 |  |  | 63 | 0.439 | 0.5047 | Yes |
| 54 | GMNN |  |  | 64 | 0.435 | 0.5120 | Yes |
| 55 | RANBP1 |  |  | 65 | 0.430 | 0.5193 | Yes |
| 56 | HIST1H1E |  |  | 66 | 0.429 | 0.5265 | Yes |
| 57 | FBXO5 |  |  | 67 | 0.420 | 0.5335 | Yes |
| 58 | CCNB1 |  |  | 68 | 0.420 | 0.5406 | Yes |
| 59 | LMNB1 |  |  | 69 | 0.417 | 0.5476 | Yes |
| 60 | CENPH |  |  | 71 | 0.412 | 0.5544 | Yes |
| 61 | RRM1 |  |  | 72 | 0.412 | 0.5613 | Yes |
| 62 | SNRPD1 |  |  | 74 | 0.408 | 0.5681 | Yes |
| 63 | LSM3 |  |  | 75 | 0.407 | 0.5749 | Yes |
| 64 | NUCKS1 |  |  | 78 | 0.404 | 0.5815 | Yes |
| 65 | H2AFV |  |  | 82 | 0.397 | 0.5878 | Yes |
| 66 | KIF4A |  |  | 84 | 0.393 | 0.5943 | Yes |
| 67 | LSM5 |  |  | 85 | 0.393 | 0.6009 | Yes |
| 68 | CENPN |  |  | 87 | 0.390 | 0.6074 | Yes |
| 69 | GTSE1 |  |  | 89 | 0.389 | 0.6138 | Yes |
| 70 | PHF19 |  |  | 93 | 0.380 | 0.6199 | Yes |
| 71 | DTYMK |  |  | 94 | 0.379 | 0.6262 | Yes |
| 72 | CDKN3 |  |  | 97 | 0.376 | 0.6323 | Yes |
| 73 | RAN |  |  | 98 | 0.375 | 0.6386 | Yes |
| 74 | DNAJC9 |  |  | 103 | 0.373 | 0.6444 | Yes |
| 75 | SPC25 |  |  | 104 | 0.372 | 0.6507 | Yes |
| 76 | CENPE |  |  | 105 | 0.372 | 0.6569 | Yes |
| 77 | FEN1 |  |  | 107 | 0.371 | 0.6631 | Yes |
| 78 | ANLN |  |  | 111 | 0.358 | 0.6688 | Yes |
| 79 | NDC80 |  |  | 115 | 0.354 | 0.6744 | Yes |
| 80 | KIF23 |  |  | 117 | 0.351 | 0.6802 | Yes |
| 81 | VRK1 |  |  | 118 | 0.350 | 0.6861 | Yes |
| 82 | DNMT1 |  |  | 121 | 0.345 | 0.6916 | Yes |
| 83 | ASPM |  |  | 122 | 0.342 | 0.6974 | Yes |
| 84 | TUBB4B |  |  | 125 | 0.340 | 0.7029 | Yes |
| 85 | CCDC34 |  |  | 127 | 0.339 | 0.7085 | Yes |
| 86 | PTGES3 |  |  | 128 | 0.336 | 0.7141 | Yes |
| 87 | SNRPB |  |  | 132 | 0.332 | 0.7194 | Yes |
| 88 | CCNA2 |  |  | 135 | 0.331 | 0.7247 | Yes |
| 89 | ANP32E |  |  | 136 | 0.330 | 0.7303 | Yes |
| 90 | USP1 |  |  | 138 | 0.329 | 0.7357 | Yes |
| 91 | MIS18A |  |  | 142 | 0.322 | 0.7408 | Yes |
| 92 | CALM2 |  |  | 143 | 0.322 | 0.7462 | Yes |
| 93 | RAD21 |  |  | 147 | 0.316 | 0.7512 | Yes |
| 94 | CDCA2 |  |  | 150 | 0.309 | 0.7561 | Yes |
| 95 | CKAP2 |  |  | 151 | 0.308 | 0.7613 | Yes |
| 96 | KIF22 |  |  | 154 | 0.306 | 0.7663 | Yes |
| 97 | SRSF7 |  |  | 155 | 0.306 | 0.7714 | Yes |
| 98 | CEP55 |  |  | 158 | 0.306 | 0.7763 | Yes |
| 99 | CDCA3 |  |  | 160 | 0.304 | 0.7813 | Yes |
| 100 | TMEM106C |  |  | 162 | 0.303 | 0.7863 | Yes |
| 101 | AURKB |  |  | 163 | 0.303 | 0.7914 | Yes |
| 102 | CMC2 |  |  | 170 | 0.297 | 0.7958 | Yes |
| 103 | MZT1 |  |  | 174 | 0.295 | 0.8004 | Yes |
| 104 | PSIP1 |  |  | 175 | 0.294 | 0.8053 | Yes |
| 105 | DDX39A |  |  | 183 | 0.293 | 0.8095 | Yes |
| 106 | NUF2 |  |  | 188 | 0.287 | 0.8139 | Yes |
| 107 | CCNB2 |  |  | 190 | 0.286 | 0.8186 | Yes |
| 108 | CDC20 |  |  | 193 | 0.284 | 0.8232 | Yes |
| 109 | DEPDC1 |  |  | 196 | 0.283 | 0.8277 | Yes |
| 110 | HMGB3 |  |  | 200 | 0.282 | 0.8321 | Yes |
| 111 | CYCS |  |  | 207 | 0.279 | 0.8362 | Yes |
| 112 | KIF20B |  |  | 208 | 0.278 | 0.8408 | Yes |
| 113 | HNRNPDL |  |  | 220 | 0.271 | 0.8442 | Yes |
| 114 | CENPA |  |  | 238 | 0.259 | 0.8467 | Yes |
| 115 | ILF2 |  |  | 240 | 0.258 | 0.8509 | Yes |
| 116 | BUB3 |  |  | 311 | 0.228 | 0.8472 | Yes |
| 117 | C12orf75 |  |  | 316 | 0.227 | 0.8506 | Yes |
| 118 | CKAP2L |  |  | 327 | 0.224 | 0.8533 | Yes |
| 119 | PLK1 |  |  | 364 | 0.213 | 0.8530 | Yes |
| 120 | DLGAP5 |  |  | 371 | 0.211 | 0.8559 | Yes |
| 121 | CENPW |  |  | 373 | 0.211 | 0.8593 | Yes |
| 122 | AURKA |  |  | 375 | 0.210 | 0.8628 | Yes |
| 123 | HAT1 |  |  | 381 | 0.209 | 0.8657 | Yes |
| 124 | TROAP |  |  | 435 | 0.193 | 0.8633 | Yes |
| 125 | EXOSC8 |  |  | 456 | 0.189 | 0.8643 | Yes |
| 126 | KNSTRN |  |  | 483 | 0.183 | 0.8646 | Yes |
| 127 | HNRNPA2B1 |  |  | 510 | 0.178 | 0.8648 | Yes |
| 128 | PRC1 |  |  | 514 | 0.178 | 0.8674 | Yes |
| 129 | HSP90AA1 |  |  | 600 | 0.164 | 0.8610 | No |
| 130 | S100B |  |  | 688 | 0.151 | 0.8542 | No |
| 131 | RNASEH2A |  |  | 1579 | 0.082 | 0.7598 | No |
| 132 | GGH |  |  | 2149 | 0.059 | 0.6996 | No |
| 133 | ARL6IP1 |  |  | 2421 | 0.050 | 0.6713 | No |
| 134 | PDLIM7 |  |  | 3015 | 0.033 | 0.6080 | No |
| 135 | TPST2 |  |  | 7315 | -0.045 | 0.1461 | No |
| 136 | MAP3K7CL |  |  | 8672 | -0.109 | 0.0020 | No |
| 137 | DLG1 |  |  | 8857 | -0.130 | -0.0156 | No |
| 138 | CYR61 |  |  | 8901 | -0.136 | -0.0179 | No |
| 139 | PLP1 |  |  | 8931 | -0.139 | -0.0187 | No |
| 140 | PTN |  |  | 8989 | -0.147 | -0.0224 | No |
| 141 | AHNAK2 |  |  | 9146 | -0.177 | -0.0362 | No |
| 142 | PRCP |  |  | 9153 | -0.178 | -0.0339 | No |
| 143 | SCRG1 |  |  | 9200 | -0.191 | -0.0356 | No |
| 144 | MIAT |  |  | 9386 | -0.332 | -0.0500 | No |
| 145 | CTGF |  |  | 9400 | -0.375 | -0.0451 | No |
| 146 | IGFBP3 |  |  | 9424 | -0.502 | -0.0391 | No |
| 147 | MALAT1 |  |  | 9436 | -0.735 | -0.0279 | No |
| 148 | IGFBP5 |  |  | 9438 | -0.797 | -0.0147 | No |
| 149 | SCG2 |  |  | 9440 | -0.879 | 0.0000 | No |
Table: GSEA details [plain text format]

  

Fig 2: FELDMAN\_2020\_G1: Random ES distribution      
 Gene set null distribution of ES for **FELDMAN\_2020\_G1**
